# Ion channel expression patterns in glioblastoma stem cells with functional and therapeutic implications for malignancy

**DOI:** 10.1371/journal.pone.0172884

**Published:** 2017-03-06

**Authors:** Julia Pollak, Karan G. Rai, Cory C. Funk, Sonali Arora, Eunjee Lee, Jun Zhu, Nathan D. Price, Patrick J. Paddison, Jan-Marino Ramirez, Robert C. Rostomily

**Affiliations:** 1 Center for Integrative Brain Research, Seattle Children's Research Institute, Seattle, Washington, United States of America; 2 Institute for Systems Biology, Seattle, Washington, United States of America; 3 Human Biology Division, Fred Hutchinson Cancer Research Center, Seattle, Washington, United States of America; 4 Department of Genetics and Genomic Sciences, Icahn Institute of Genomics and Multiscale Biology, Icahn School of Medicine at Mount Sinai, New York, New York, United States of America; 5 Department of Hematology and Medical Oncology, The Tisch Cancer Institute, Icahn School of Medicine at Mount Sinai, New York, New York, United States of America; 6 Department of Neurosurgery, University of Washington, Seattle, Washington, United States of America; 7 Institute for Stem Cell and Regenerative Medicine, University of Washington, Seattle, Washington, United States of America; 8 Houston Methodist Research Institute, Houston, Texas, United States of America; 9 Department of Neurosurgery, Houston Methodist Hospital, Houston, Texas, United States of America; Swedish Neuroscience Institute, UNITED STATES

## Abstract

Ion channels and transporters have increasingly recognized roles in cancer progression through the regulation of cell proliferation, migration, and death. Glioblastoma stem-like cells (GSCs) are a source of tumor formation and recurrence in glioblastoma multiforme, a highly aggressive brain cancer, suggesting that ion channel expression may be perturbed in this population. However, little is known about the expression and functional relevance of ion channels that may contribute to GSC malignancy. Using RNA sequencing, we assessed the enrichment of ion channels in GSC isolates and non-tumor neural cell types. We identified a unique set of GSC-enriched ion channels using differential expression analysis that is also associated with distinct gene mutation signatures. In support of potential clinical relevance, expression of selected GSC-enriched ion channels evaluated in human glioblastoma databases of The Cancer Genome Atlas and Ivy Glioblastoma Atlas Project correlated with patient survival times. Finally, genetic knockdown as well as pharmacological inhibition of individual or classes of GSC-enriched ion channels constrained growth of GSCs compared to normal neural stem cells. This first-in-kind global examination characterizes ion channels enriched in GSCs and explores their potential clinical relevance to glioblastoma molecular subtypes, gene mutations, survival outcomes, regional tumor expression, and experimental responses to loss-of-function. Together, the data support the potential biological and therapeutic impact of ion channels on GSC malignancy and provide strong rationale for further examination of their mechanistic and therapeutic importance.

## Introduction

Glioblastoma multiforme (GBM; grade IV glioma) is the most prevalent and malignant form of primary brain tumor in adults [1,2]. Median survival is a mere 15 months despite radiotherapy, surgical resection, and chemotherapeutic interventions [1]. GBM tumors are especially difficult to treat, since surgical resection invariably leaves behind glioblastoma stem-like cells (GSCs), which are highly invasive tumor cells uniquely resistant to standard therapies.

GSCs are a population of GBM cells that play a major role in the particularly aggressive nature of GBM tumors and share traits with neural stem cells (NSCs), including self-renewal and multipotency [3]. Remarkably, transplantation of only 100 GSCs into the mouse forebrain is sufficient to form a glioma tumor [4]. Several features of GSCs contribute to GBM malignancy following initial tumor formation, including rapid proliferation and highly diffuse invasion throughout the brain [5]. Additionally, standard chemotherapeutic agents, which eradicate the majority of GBM cells, have a reduced effect on GSCs, and surviving GSCs contribute to tumor recurrence, a hallmark of GBM [5,6]. These features underscore the necessity for development of novel therapeutic candidates that precisely target GSCs and halt uncontrolled growth and invasion.

Ion channels passively conduct ions down their electrochemical gradient in response to external stimuli, whereas ion transporters use energy to pump ions across their concentration gradients [7,8]. Ion channels and pumps are responsible for conducting electrical currents in all nerve, muscle, and cardiac cells, however, they also play vital roles outside of regulating electrical excitability in both normal and cancerous cells. It is increasingly being understood that dysregulated ion channels and pumps are implicated in multiple processes in various cancers [9], including regulation of the cell cycle [10], migration [11], and apoptosis [12]. Promisingly, inhibitors to various ion channels have been demonstrated to hinder tumor formation and growth [13,14].

Ion channels and transporters are likewise implicated in GBM tumor growth and malignancy [15–17]. Genomic analysis reveals that genes involved in passing or transporting Na^+^, K^+^, and Ca^2+^ are among the most frequently mutated functional groups in GBM affecting 90% of the GBM samples studied [18,19]. Functionally, ion channels and pumps influence both GBM cell migration and proliferation. For instance, dysregulated K^+^ and Cl^-^ channels regulate osmotic drive allowing for cell shape and volume changes that promote glioma cell migration [20], and Ca^2+^-activated K^+^ (BK) channels control glioma cell growth [21]. However, little is known about the expression and functional relevance of ion channels in the stem cell population despite their central importance to GBM tumor initiation and progression.

We propose that dysregulation of ion channel expression is central to the abnormal growth and migratory properties that drive GSC malignancy. Therefore, a greater understanding of the ion channels operating in GSCs may reveal novel, therapeutically relevant mechanisms to target GSCs. To assess the expression pattern of ion channels that may contribute to glioma malignancy, we analyzed an RNA sequencing database of 20 patient-derived GSC isolates and 5 neural cell type controls. We identified a unique set of druggable ion channels enriched in GSCs that were associated with distinct gene mutation signatures and poor patient survival outcomes. Pharmacological blockade and genetic knockdown of these channels impaired GSC viability. Identification of GSC-enriched ion channels and the mechanism by which they drive GSC malignancy could identify novel therapeutics to inhibit GSC-driven tumor growth and improve patient outcomes.

## Results

### Expression of ion channels, transporters, and gap junctions in GSCs

To profile the enrichment patterns of ion channel genes in GSCs, we compared transcriptomic data for 20 human GSC isolates to that of 3 human NSC lines and 2 normal human astrocyte (NHA) cell lines. We used a comprehensive list of 266 (7 out of the original 273 were not available in our dataset) druggable human ion channel genes [22] (guidetopharmacology.org) and 152 human ion transporter genes (broadinstitute.org/gsea/msigdb, GO:0015075). The strategy of comparing GSCs to NSCs/NHAs was used as a first approximation to enrich for genes specific to malignant stem cell phenotypes and not shared by non-transformed neural progenitors or astrocytic glia. Gene expression was calculated as CPM (counts per million) values for this data analysis. Note that, as opposed to CPM, other reports referred to later used FPKM (fragments per kilobase of exon per million reads mapped) values in a similar way to quantify and report differential gene expression. Additional details are provided for: cell lines (S1 Table), RNA-seq methods (Materials and methods), and ion channel gene set (S2 Table). S1 Fig summarizes the experimental design used throughout this study.

**Fig 1:**
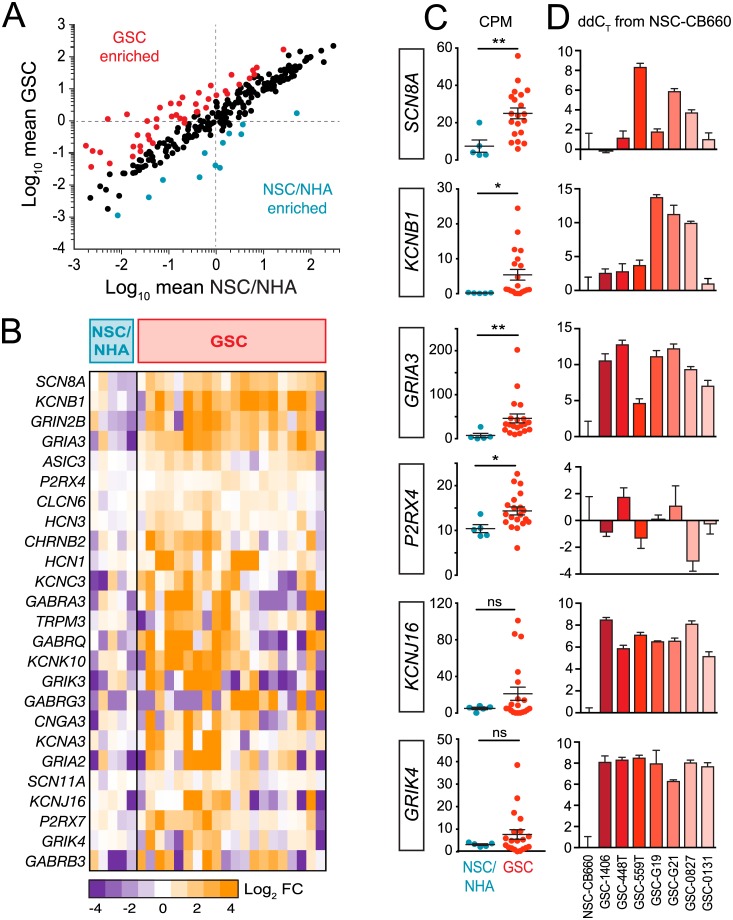
RNA sequencing identifies ion channels enriched in GSCs. A. Mean GSC CPM values plotted against mean control (NSC and NHA) CPM values for all ion channel-related genes. Red and blue points represent genes that are differentially expressed ≥|2| log_2_ fold change in each subclass. B. Heat map of the most differentially enriched ion channels in GSCs compared to control NSCs and NHAs by GSEA analysis. Each column represents log_2_ fold-change values (compared to averaged values across NSCs/NHAs) from a distinct cell isolate after averaging triplicate CPM values. Ion channels for which average GSC CPM values were <1 were not included, and individual CPM values of 0 were replaced with 0.01. C. CPM values for six of the most differentially enriched genes shown in panel (B). Bars, mean ± SEM. Mann-Whitney test; *p<0.05, **p<0.01, ns = not significant. D. Real-time qPCR analysis of a selected number of IGCs in several GSC isolates. C_T_ values were normalized to *ACTB* (β-Actin) C_T_ values; ddC_T_ values relative to NSC-CB660 are shown. Bars, mean ± stdev. N = 3. GSC, Glioblastoma stem-like cells; NSC, human fetal neural stem cells including c-myc immortalized from cortex (NSC-CX) and brainstem (NSC-VM); NHA, normal human astrocytes including RasV12 transformed (NHA-RAS).

Using a simple fold-change approach, we found differential expression (≥|2| log_2_ fold change) in 56 out of 251 ion channel-related genes (15 genes were excluded due to zero values in denominator) when comparing GSCs to NSCs/NHAs. Of these 56 genes, 44 were GSC-enriched (≥2 log_2_ fold change; Fig 1A, red points), and 12 were NSC/NHA-enriched (≤-2 log_2_ fold change; Fig 1A, blue points). Since fold change differences can skew contributions from lowly expressed genes, Gene Set Enrichment Analysis (GSEA; details in Materials and methods) was used to further analyze differential expression patterns. Using a Signal2Noise metric, which accounts for both mean absolute levels and variance within the classes, we found that 107 out of 266 ion channels highly contributed to the enrichment of ion channels in GSCs compared to control cells. These genes were rank-ordered according to their GSEA enrichment score, and the top 40 differentially expressed ion channel-related genes were selected for further study. Twenty-five of these met our threshold expression criteria (mean GSC CPM ≥1) and formed the basis of a GSC-enriched ion channel gene set (hereby referred to as “IGCs”) used for all remaining analyses in this study (Fig 1B; see S1 Fig for summary of selection criteria). Notably, these genes represented a diversity of ion channel types. Scatter plots of CPM values for selected highly-ranked IGCs demonstrated the significant differences in expression between GSC and NSC/NHA cell lines (Fig 1C). This was validated in multiple GSC lines compared to the NSC line CB660 using real-time quantitative PCR (RT-qPCR) for all IGCs tested except *P2RX4*, for which expression differences were not robust (Fig 1D). In summary, we found a high frequency (~40%) of ion channel genes associated with GSCs compared to control NSCs/NHAs, and ~10% overall formed the basis of a GSC-enriched ion channel gene set.

By contrast, ion transporters, which also contribute to cancer progression in multiple cell types [23], were infrequently enriched in GSCs. Of the 152 ion transporters assessed, only 1 (~0.7%) met our criteria to be considered differentially enriched (average CPM values ≥1 and ≥2 log_2_ fold change enriched in GSCs vs. NSCs/NHAs; data not shown). Gap junction proteins also modulate electrical properties of cells and have a recently demonstrated role in promoting malignant phenotypes in GBM [24]. Gap junction-forming connexins, which were included in the overall set of 266 ion channel genes, were enriched in some GSC isolates (S2 Fig), but their low absolute expression levels failed to meet our cutoff for further analysis. In summary, when comparing GSCs to other neural cell types, differential expression of ion channel genes is markedly more prevalent than that of ion transporter and connexin genes. Based on these observations, our subsequent analyses focused on the potential importance of this selected cohort of 25 IGCs (see S4 Table for summary of IGCs).

### IGC expression in normal neural cells

The therapeutic relevance of IGCs as drug targets in GBM may be limited by their expression in other normal tissues and neural cell types not included in the primary enrichment strategy described above. Therefore, we examined their expression in public databases of normal tissues and other neural cell types. Among the top IGCs of interest, the majority (16/25) were appreciably expressed in brain compared to other tissues (gtexportal.org; data not shown), 6/25 were more highly expressed in one or multiple other tissue types compared to brain, and 3/25 were not tissue-specific. To examine IGC expression in brain-specific cell subpopulations, we analyzed expression of IGCs in an RNA-seq database of isolated cortical human neural cell types, including astrocytes (fetal, adult, and reactive), neurons, oligodendrocytes, microglia, and endothelial cells [25] (S3 Fig). Out of our 25 previously identified IGCs, 1 was not available in this database, and 3 were expressed at levels below the arbitrary threshold used for selection of the IGC gene set (mean FPKM ≥1 in at least one cell type). Of the 21 remaining IGCs, 10 were specifically enriched in neurons (*GRIA2*, *GRIA3*, *GABRB3*, *GABRA3*, *KCNB1*, *KCNA3*, *SCN8A*, *GRIN2B*, *HCN1*, and *GABRG3*), 5 in astrocytes (*KCNJ16*, *CLCN6*, *TRPM3*, *GRIK3*, and *CNGA3*), and 1 in microglia (*P2RX4*), while the remaining 5 (*P2RX7*, *KCNK10*, *HCN3*, *GABRQ*, and *CHRN2B*) were expressed across multiple cell types. As noted above, 3 of the genes in the IGC set of 25 (*KCNC3*, *SCN11A*, and *GRIK4*) were expressed at very low levels (mean subclass FPKM <1) in all normal neural cell classes (S3 Fig, “Low abundance”), which may suggest that these IGCs are specific to cancerous cell types. This analysis identified non-cancerous tissue- and cell type-specific IGC expression patterns that could be important considerations for selecting IGCs as potential therapeutic targets for GBM. The varied patterns in IGC expression suggest that each IGC may possess a unique profile of systemic and neural toxicity that must be considered when targeting IGCs.

### IGC expression is associated with GBM molecular classification

**Fig 2:**
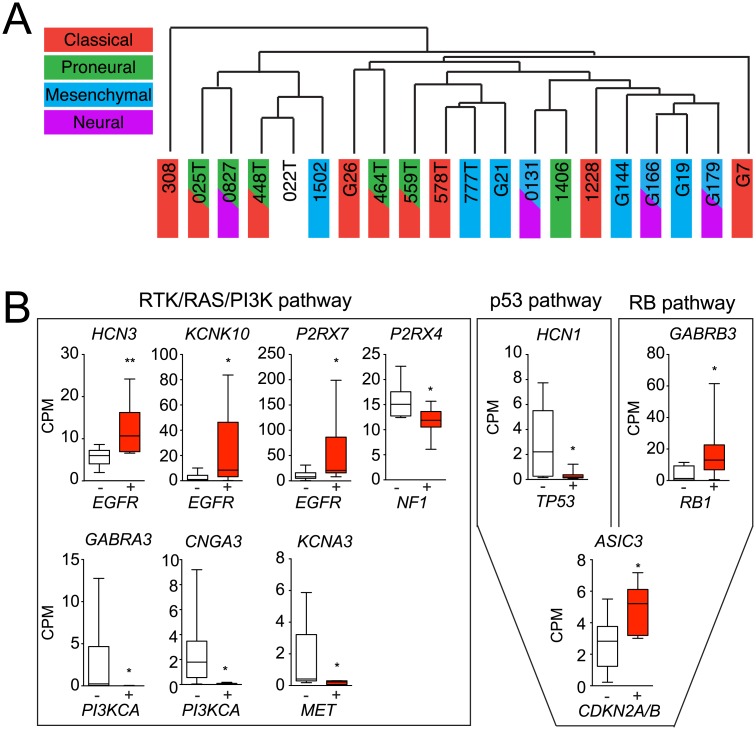
GSC-enriched ion channels are associated with GBM molecular features. A. Hierarchical clustering of GSCs by molecular subtype based on CPM values of all 266 ion channels. B. Correlation of IGCs with gene mutation signatures of critical GBM signaling pathways (see S1 Table for details). Bars, mean ± min/max, IQR. Two-tailed Mann-Whitney test; *p<0.05, **p<0.01.

To better understand the potential clinical relevance of IGC expression, we characterized correlations between IGC expression and GBM molecular subtypes (Classical, Mesenchymal, Neural, or Proneural) [26], as well as driver genetic mutations in the GSCs. Molecular subtype classifications were previously assigned to GSC isolates (S1 Table) [27]. Unsupervised hierarchical clustering was performed based on CPM values of the entire set of 266 ion channels. Based on ion channel expression alone, GSC isolates of the same molecular subtype generally clustered together (Fig 2A), suggesting that ion channel expression patterns segregate with molecular subtypes.

We next investigated the association of IGCs with well-characterized gene mutations in three critical GBM signaling pathways: RTK/RAS/PI(3)K, p53, and RB [28]. Exome sequencing was carried out on GSCs to reveal gene mutations in these pathways (S1 Table), and direct associations between IGC expression and gene mutations were identified (Fig 2B). The majority of associations were between IGCs and mutations in the RTK/RAS/PI(3)K pathway (*EGFR*, *PI3KCA*, *NF1*, *MET*), which can regulate proliferation and survival [28]. Fewer associations were observed in the RB pathway (*RB1*, *CDKN2A/B*), responsible for regulating G1/S progression, and the p53 pathway (*TP53*, *CDKN2A/B*), which regulates senescence and apoptosis. These preliminary findings suggest associations between IGC expression and clinically relevant GBM subtypes as well as potential functional interactions between specific IGCs and mutation-driven oncogenic signaling pathways that may prove to have prognostic or therapeutic value. However, further prospective studies with larger sample sizes and routine standardized molecular analyses are required for validation.

### IGC expression predicts GBM patient survival

**Fig 3:**
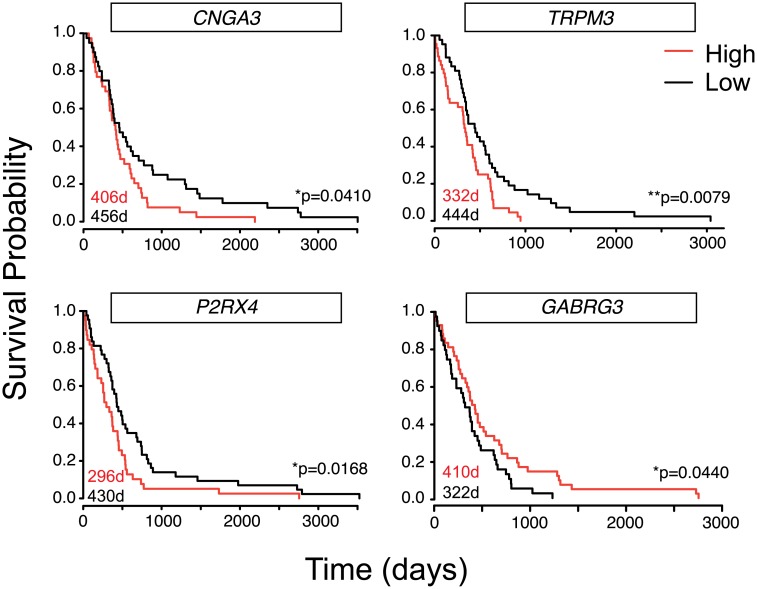
IGCs are associated with poor clinical outcomes. Expression levels of IGCs were identified from 525 TCGA bulk GBM microarray expression samples. Samples for which expression levels were highest and lowest (top and bottom 10%) were then compared for time to death. Median days to death for each group reported on graph.

To test the prognostic significance of selected IGCs, we determined the correlation between their expression levels and patient survival using The Cancer Genome Atlas (TCGA) human GBM microarray database (n = 525; https://tcga-data.nci.nih.gov/tcga/). While the lack of paired normal samples precluded a determination of IGC enrichment in GBM versus normal brain tissue, analysis of IGC expression did reveal correlations with patient outcomes. Survival of patient cohorts was quantitated using Kaplan-Meier analysis and stratified by high (top 10%) or low (bottom 10%) IGC expression for the top 25 IGCs. Using this approach, four IGCs were found to have significant associations with survival. High expression of *CNGA3*, *TRPM3*, and *P2RX4* was associated with significantly shorter median survival times, while high expression of *GABRG3* predicted longer survival times (Fig 3). The divergent associations between IGC expression and survival suggest the possibility that individual IGCs may function to either promote or inhibit malignancy. Since TCGA expression data derives from bulk GBM tissue samples, further studies are warranted in GSC models to determine whether associations between IGC expression and GBM malignancy can be attributed to GSC-specific function.

### IGCs are enriched in distinct GBM tumor regions

**Fig 4:**
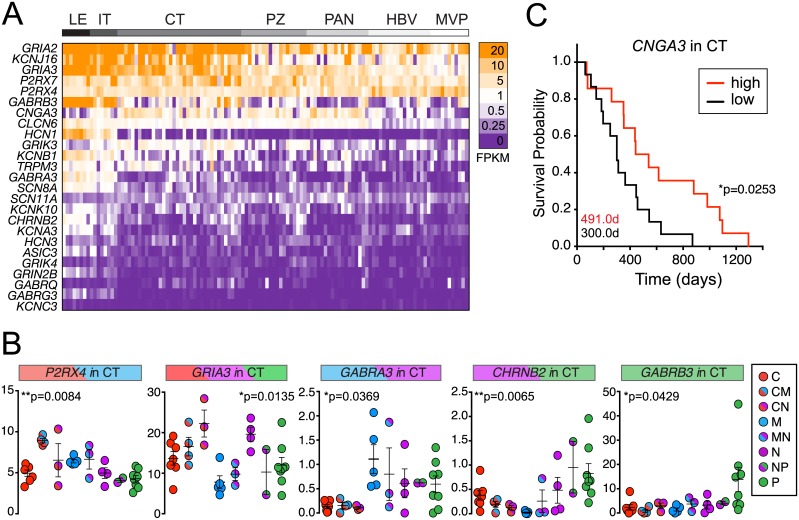
IGCs are enriched in distinct GBM tumor regions. A. Heat map of expression levels of the top 25 IGCs in various GBM tumor regions from the Ivy GAP RNA-seq database. B. IGCs associated with particular molecular subtypes in CT samples. Colored headers indicate predominant subtype associated with that ion channel. Bars, mean ± SEM. Kruskal-Wallis test across molecular subtypes. C. Survival curve for *CNGA3* within CT samples stratified high/low by median FPKM value (3.908). Log-rank (Mantel-Cox) test. Median days to death for each group reported on graph. Leading Edge (LE), Infiltrating Tumor (IT), Cellular Tumor (CT), Perinecrotic zone (PZ), Pseudopalisading cells around necrosis (PAN), Hyperplastic blood vessels in cellular tumor (HBV), Microvascular Proliferation (MVP).

To determine whether IGCs are regionally expressed in unique histological domains of GBM tumors with functional and clinical relevance, we examined IGC expression patterns in the Ivy Glioblastoma Atlas Project (Ivy GAP) database [29]. The Ivy GAP database dissects specific anatomic tumor regions that include the leading edge (LE) (with few tumor cells), infiltrating tumor (IT), cellular (central solid) tumor (CT), necrotic zones (PAN/PZ), and vascular regions (HBV/MVP). Overall, 13 of 25 IGCs were either not expressed or detected at low levels (<1 FPKM across all anatomic regions). When considering relative differences in expression by location, the greatest number of IGCs (18/25) were enriched in the LE and IT compartments compared to other regions (Fig 4A; ≥2 fold-change of mean LE/IT vs. mean all other regions). This was reflective of a larger trend, whereby half of all ion channels (131/273) were ≥2-fold higher at the tumor edge (LE/IT). Since tumor cell densities are negligible in LE and low in IT, IGC enrichment in LE/IT likely reflects IGC expression in normal neural cell types (neurons, microglia, etc.), which were not included in the original RNA-seq screen to identify IGCs.

We reasoned that increased absolute expression levels in different histological regions of GBM could also identify IGCs with potential clinical and functional relevance. Overall, 9/25 IGCs were consistently expressed at ≥5 FPKM in at least one anatomic region (Fig 4A). Nearly all of the samples from the LE/IT and CT regions demonstrated this level of expression for the nine IGCs, while lower and more variable expression was observed in samples from necrotic (PZ/PAN) and vascular (HBV/MVP) features. For example, in the HBV/MVP vascular regions, five of these nine IGCs (*GRIA2*, *KCNJ16*, *GRIA3*, *P2RX7*, and *P2RX4*) were expressed at appreciable levels (≥5 FPKM) in a majority of samples. IGC expression in the vascular compartment is consistent with the known role of GBM vasculature to provide a supportive niche for GSCs [30,31]. However, since GSCs are a minority cell population and IGC expression may overlap with other cell types (see S3 Fig), the specificity of IGC expression for GSC localization requires additional detailed studies.

We next examined how IGC expression levels in the Ivy GAP database correlated with molecular subtypes and survival. We chose to study this in the solid CT region only, since this region forms the bulk of GBM tumors and comprised the largest number of samples for any of the anatomic subsets. Among all 25 IGCs studied that were expressed at appreciable levels (average FPKM across CT >1), five were significantly associated with particular molecular subtypes in CT (*P2RX4*, *GRIA3*, *GABRA3*, *CHRNB2*, and *GABRB3*; Fig 4B). We also examined whether IGC expression within the CT region was associated with prolonged or reduced survival in these patients. Kaplan-Meier plots were generated for IGCs with median CT FPKM levels >1 (9/25) that compared survival times in patient samples with high (above median FPKM) or low (below median FPKM) IGC levels. Samples with low levels of *CNGA3* were associated with significantly reduced survival rates (Fig 4C). Significant differences in survival rates were not observed between molecular subtypes overall (data not shown), indicating that the survival association with *CNGA3* is not an artifact of the disproportionate number of subtype-specific samples with high *CNGA3* expression.

### Pharmacological blockade of ion channels restricts GSC viability

**Fig 5:**
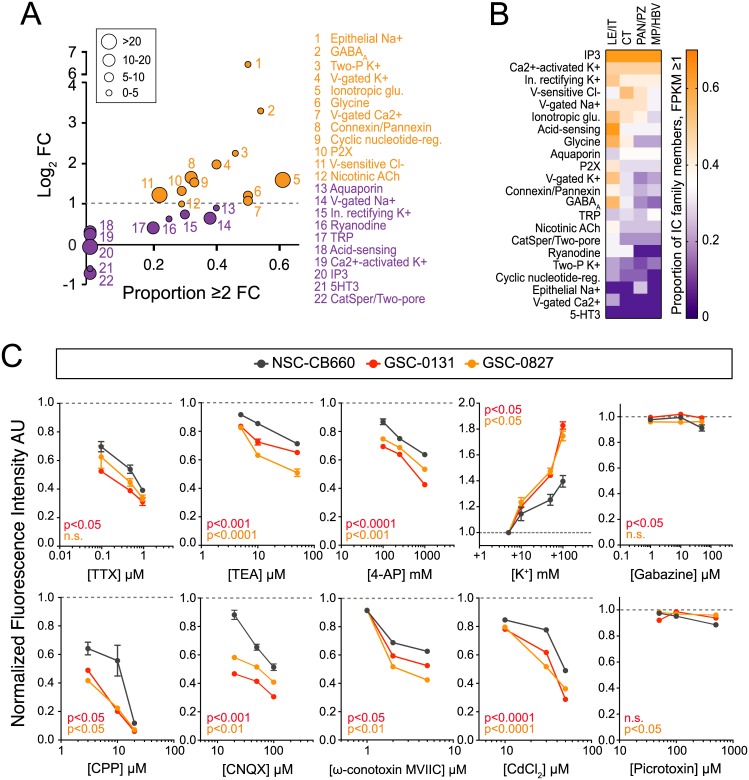
Ion channel blockade reduces GSC viability. A. Summary of functionally-related ion channel families enriched in GSCs (orange) compared to controls. Y-axis, mean log_2_ fold change (GSC vs. NSC/NHA) values for each ion channel family; x-axis, proportion of ion channel family members > 2-fold change (GSC vs. NSC/NHA); bubble size corresponds to average GSC CPM value. Genes were excluded if average NSC/NHA values were zero. Families were considered if they contained more than one member. B. Proportion of ion channel family members expressed at >1 FPKM within distinct GBM regional compartments as revealed by Ivy GAP analysis (only families with at least three members are shown). C. Antagonists for top IGC families and selected specific ion channel blockers were applied to GSC-0827, GSC-0131, and NSC-CB660 in media at indicated concentrations. MTT viability assay was performed at 72 hours. Fluorescence arbitrary units (AU) were averaged across triplicates and normalized to control media conditions (dashed line at 1.0). Bars, mean ± SEM. N = 3–4. Repeated measures two-way ANOVA with Dunnett’s multiple comparison test compared to NSC-CB660. n.s., not significant.

Thus far, we have identified individual GSC-enriched ion channels, which are members of larger ion channel families with overlapping functions. However, the majority of available drugs target the broader functionality of these families rather than specific channels. Therefore, to explore the potential therapeutic relevance of targeting IGCs, we assessed GSC enrichment of classes of functionally related ion channels. We reasoned that this approach would mitigate the potential limitations imposed by ion channel redundancy. We found that 12 out of 22 ion channel families were enriched more than 2-fold in GSCs compared to NSC/NHA controls, and all 12 of these families also contained a high proportion of IGCs (at least 1/5 of members enriched >2-fold) (Fig 5A, orange points). The most highly enriched families (>5-fold) were epithelial Na^+^ channels and GABA_A_ receptors. Fold-change values, however, are likely to be skewed by contributions from rare, yet highly enriched ion channels. Therefore, it is also important to consider the proportion of family members enriched, as we have here; the ionotropic glutamate receptor family had the highest proportion of enriched members (11/18). We also examined regional patterns of ion channel family expression from the Ivy GAP dataset (Fig 5B; S4 Fig). A large proportion of IP3 receptors, Ca^2+^-activated K^+^ channels, and inwardly-rectifying K^+^ channels were highly enriched across all tumor regions. Once again, several ion channel classes were highly enriched at the tumor leading edge with decreasing levels of expression towards HBV/MVP areas.

To test whether IGC-related families are functionally relevant to the malignant properties of GSCs, we examined whether ion channel blockers inhibit GSC growth in vitro. Pharmacological blockade has advantages over genetic knockdown/loss-of-function studies, since the action of similarly functioning ion channels can be blocked while avoiding compensation by alternative channels [32]. An MTT viability assay was performed for large-scale, rapid viability assessment. Several drugs were tested based on the enrichment of their channel targets in the GSC RNA-seq dataset (S4 Table; see Materials and methods for drug details). GSC lines -0827 and -0131 were selected for in vitro pharmacological evaluation of viability due to their rapid growth rates in vitro, tumorigenic capacity in vivo [33], and extensive molecular characterization [27]. Compounds were added at increasing concentrations to cell isolates, and viability was measured 72 hours later. Increasing doses of TTX, TEA, 4-AP, CPP, CNQX, ω-Conotoxin MVIIC, and CdCl_2_ dramatically reduced cell viability across all lines, while K^+^ enhanced viability in a dose-dependent manner (Fig 5C). GSC lines -0827 and -0131 were more sensitive to these effects than NSC-CB660 for all compounds tested. Neither gabazine nor picrotoxin (GABA_A_ receptor blockers) affected GSC or NSC viability. These results demonstrate the functional relevance of K^+^ channels, voltage-gated Na^+^ channels, voltage-gated Ca^2+^ channels, voltage-gated Cl^-^ channels, and ionotropic glutamate receptors to GSC viability as predicted by GSC enrichment of IGC families or individual members.

### *SCN8A*, *KCNB1*, or *GRIA3* knockdown reduces GSC viability

**Fig 6:**
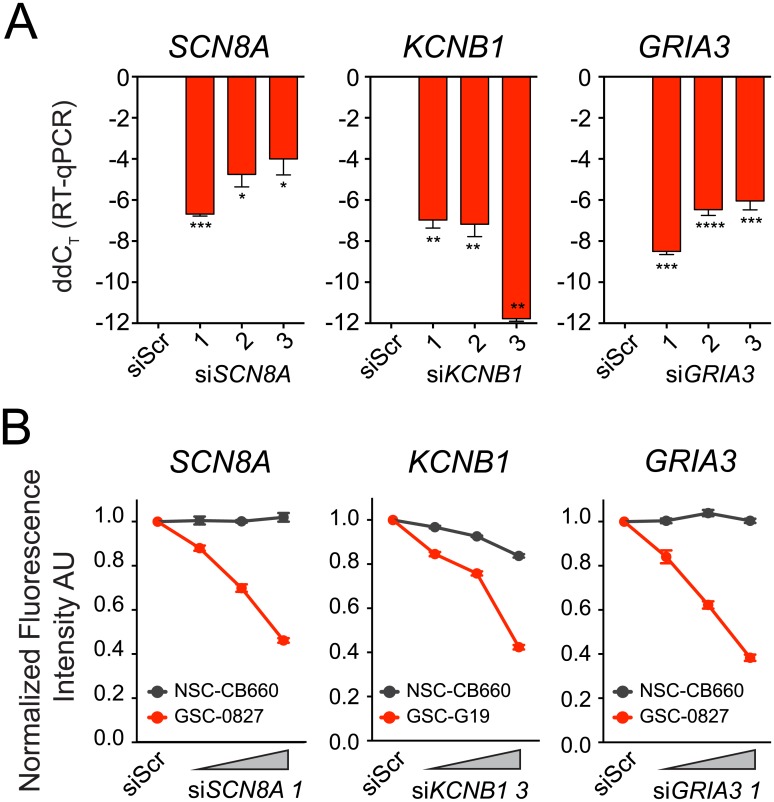
siRNA-mediated knockdown of selected IGCs impairs GSC viability. A. Real-time qPCR analysis of IGC expression levels 24 hours after transfection of scrambled negative control siRNA (siScr) and individual siRNA candidates (1, 2, 3) for each IGC. C_T_ values were normalized to *ACTB* (β-Actin) C_T_ values; ddC_T_ from siScr. Bars, mean ± stdev. N = 3. One-way ANOVA with Dunnet’s post-test compared to siScr, *p<0.05, **p<0.01, ***p<0.001, ****p<0.0001. B. CellTiter-Glo viability assay 72 hours following siRNA-mediated knockdown of IGCs. GSC viability decreased in response to increasing amounts of siRNA (2, 5, and 20 pmol). Fluorescence arbitrary units (AU) were normalized to siScr levels. Bars, mean ± SEM. N = 3–4. Two-way ANOVA with repeated measures; *SCN8A*: cell type p = 0.0001, dose p<0.0001, interaction p<0.0001; *KCNB1*: cell type p = 0.0042, dose p<0.0001, interaction p<0.0001; *GRIA3*: cell type p = 0.0011, dose p<0.0001, interaction p<0.0001.

To establish the potential functional importance of specific IGCs, as opposed to broader classes of ion channels as tested above, we quantified the effects of siRNA-mediated gene expression knockdown of three IGCs, *SCN8A*, *KCNB1*, and *GRIA3*. These IGCs were selected because they were among the most highly implicated in GSC enrichment and blocking their activity with broadly-acting drugs reduced GSC viability. Three unique siRNAs for each IGC were transiently transfected into GSC lines that had high expression of the corresponding IGC (*SCN8A*, GSC-0827; *KCNB1*, GSC-G19; *GRIA3*, GSC-0827). Robust knockdown was achieved for all nine siRNA candidates (Fig 6A). The most effective siRNA candidate for each IGC, along with a scrambled siRNA control (siScr), was tested for its effects on viability, and all siRNAs demonstrated a dose response for GSC growth inhibition 72 hours after transfection (Fig 6B). At the highest dose (20 pmol per well), growth of each GSC line was inhibited 55–62% compared to siScr controls. By comparison, the NSC line CB660, which had low baseline levels of *SCN8A*, *KCNB1*, and *GRIA3* by RT-qPCR (data not shown), was inhibited only 0–16% at the highest siRNA dose. These results suggest that in addition to the broad pharmacological targeting of ion channel classes in GSCs, inhibition of specific IGCs may also have therapeutic relevance.

## Discussion

The inevitable treatment failures of GBM, the most malignant and common adult human brain tumor, are largely established by the phenotypes of its resident cancer stem cells or GSCs. By virtue of their unique propensities for self-renewal, multipotent differentiation, invasiveness, and treatment resistance, GSCs drive GBM formation and progression [4]. Therefore, a better understanding of the mechanisms that regulate GSC intrinsic physiology is expected to yield clinical benefits and is lacking in our current understanding. Ion channels are increasingly recognized as regulators of the malignant phenotypes of cancer cells [9], but have not yet been leveraged as therapeutic targets in GSCs. This is due in large part to an incomplete understanding of their GSC-specific expression patterns compared with non-cancerous neural cells. Here, we identified GSC-enriched ion channels that correlated with molecular and clinical features of GBM. Furthermore, pharmacologic blockade or genetic knockdown of IGCs differentially inhibited GSC growth compared to that of normal NSCs. Together, these data strongly support potential functional and therapeutic roles for IGCs in GBM.

This study identified a set of 25 ion channels that were highly expressed by GSCs compared to normal neural cells and were representative of many different ion channel subfamilies. This was consistent with a recent RNA-seq study that reported a collection of 18 ion channel genes identified as a molecular signature of glioma [34]. These genes were dysregulated in high grade glioma, associated with poorer survival, and representative of many different types of ion channel classes; however, there was little overlap of individual ion channels between their study and ours, likely because our study focused on ion channels unique to glioma stem cells rather than bulk tumor cells. When our study results are compiled (S5 Fig), several IGC candidates emerge as consistently implicated in contributing to GSC malignancy, among which are *SCN8A*, *KCNB1*, and *GRIA3*. One of the most reliably involved IGCs in our study is *SCN8A* (Nav1.6), a voltage-gated Na^+^ channel. Voltage-gated Na^+^ channel isoforms are aberrantly expressed in cancer cells contributing to metastatic behaviors [35,36], and Na^+^ channel mutations have been estimated in at least 90% of GBM samples [19], suggesting that they play a role in GBM malignancy. Several classes of K^+^ channels were also implicated in this study, including inwardly-rectifying, voltage-gated, and two-pore-domain K^+^ channels. These classes have been well-studied in cancer [37] and tied to GBM malignancy [38]. One candidate of particular interest is the voltage-gated K^+^ channel *KCNB1* (Kv2.1), which was expressed at high levels in GSCs and contributed to reduced GSC viability when knocked-down. mRNA knockdown of all three of these IGCs, *SCN8A*, *KCNB1*, and *GRIA3*, reduced GSC viability in our study, suggesting their importance to GSC malignancy and therapeutic potential.

One of the goals of this work was to identify ion channels that are uniquely expressed by GSCs and avoid those expressed by other cell types; the rationale of this strategy was that increased selectivity of expression in GSCs might translate to less toxic off-target effects in the therapeutic clinical setting. Along with NSCs/NHAs, we also observed differences in GSC gene expression compared to bulk GBM samples and other normal neural cell types. However, we found that many IGCs were expressed in several other neural cell populations. Across ion channel families, the majority of ionotropic glutamate and glycine receptors were enriched in both GSCs and bulk GBM samples, while 5-HT3 receptors, TRP channels, CatSper/two-pore channels, and ryanodine receptors were consistently low in both populations. GSC-specific families included GABA_A_ receptors, epithelial Na^+^, voltage-gated Ca^2+^, and two-pore domain K^+^ channels. At the individual level, several IGCs that were highly enriched in GSCs had very low or absent expression in bulk GBM tumor samples (e.g. *GRIK4*, *SCN8A*, *KCNC3*, *ASIC3*, and *HCN3*), suggesting that these candidates may be the most therapeutically tractable for specifically targeting GSCs within the tumor. Alternatively, the low expression may indicate that GSCs grown in vitro may not reflect true expression levels in vivo or that IGC expression may be obscured by the low frequency of GSCs in bulk tumor. Several of these candidates, *KCNC3*, *SCN11A*, and *GRIK4*, also failed to show appreciable expression in any normal neural cell populations (S3 Fig), which may be advantageous for precise clinical targeting of GSCs.

IGCs were also associated with distinct clinical features in this study, including GBM subtype and critical oncogenic genomic mutations. Across all 266 ion channels, there was a trend for GSCs to stratify by molecular subtype. This suggests that ion channels generally correlate with clinical features, although batch effects in GSC source origination could account for some of these effects. In line with this, IGC expression was also associated with altered clinical outcomes. This is consistent with other reports; for instance, Na^+^ channel mutations in GBM tumors have been correlated to poorer survival outcomes [19]. When considering the extremes of expression in the TCGA database, higher IGC expression of three genes (*CNGA3*, *TRPM3*, and *P2RX4*) correlated with decreased survival while higher expression of one (*GABRG3*) correlated with increased survival. By contrast, when considering expression restricted to the solid tumor (CT) region in the Ivy GAP database, high *CNGA3* was associated with increased, rather than decreased, survival as noted in TCGA. This discrepancy may reflect the complexity of ion channel functions in tumor and tumor-associated stromal cells, as well as differences in sample composition between databases and bias introduced by restricting the Ivy GAP analysis to the CT region.

A challenging problem of solid tumor biology is understanding the regional and cell type-specific variations in expression and function. We observed interesting patterns in IGC expression in GBM anatomical regions within the Ivy GAP database. Although we expected to find IGCs in the putative stem cell niche associated with the vasculature, many IGCs were highly expressed at the leading edge of the tumor instead (including many additional ion channels not shown). This likely reflects contamination from surrounding normal neural cells expressing these ICs but could be due to the presence of stem cells residing at the tumor edge and/or ion channels that are preferentially upregulated at the tumor edge for communication with the normal brain surround. Much more work will be needed to understand the regional and cell-type heterogeneity of IGCs within the tumor.

One of the major findings from this work was that both pharmacological blockade and RNA knockdown of IGCs inhibited GSC growth, which may implicate new pathways for therapeutic targeting of GBM. There is a clear and dire need for novel molecular targets in this arena. Ion channel and transporter targeting drugs are currently used to treat a variety of clinical conditions and represented over 13% of FDA-approved drugs in 2006—the second largest class of existing drugs [39]. A variety of ion channel blockers have been used to target various cancers in pre-clinical animal models [13,14]. Compellingly, anti-epileptic drugs that target voltage-gated Na^+^ channels inhibit metastatic behaviors in several cancer cell types [36], suggesting that these channels may be practical therapeutic targets in GBM. Clinically, targeting ion channels within brain tumors has many challenges associated with it. First, there is considerable functional redundancy among ion channel classes, which may mean that broad channel antagonists are needed to meaningfully impact malignant phenotypes. However, the lack of specificity of these drugs may result in profound off-target effects and deleterious side effects. Ion channels also play many crucial functions in normal surrounding neural cells, and so methods for precise targeting of ion channel-harboring brain tumor cells is needed. We have made attempts in this study to distinguish ion channels enriched specifically in GSCs compared to normal neural cell types, yet many identified IGCs are expressed in normal neural cell types. Despite these caveats, our results indicate that GBM research may be poised for novel ion channel drug discovery, and our findings offer a tractable starting point.

There were several limitations of this study. First, we compared ion channels expressed in GSCs to NSC and NHA lines in vitro to understand GSC-specific ion channel expression. While this is a good foundation for understanding GSC-specific enrichment, many control neural cell types were not included in this analysis. Lowly abundant genes were also ignored in our analyses, however it is not known how absolute CPM expression levels translate to biological processes, and it is conceivable that low CPM levels are still meaningful. We also examined expression patterns in isolated GSC lines maintained over several passages, however, GSCs normally interact with other cell types in the tumor microenvironment, which are likely to influence ion channel expression and function. Ion channel dysregulation was also monitored at the mRNA level, however, other cellular processes could modulate ion channel function and contribute to GSC malignancy. Two well-studied examples of dysregulated, post-translational cellular processes in glioma include the mislocalization of ion channels [40] and changes to ion channel sensitivity [41]. Epigenetic dysregulation is likely to play a role in glioma malignancy as well, since aberrant DNA methylation has been linked to multiple cancers [42,43]. Additionally, ion channel expression can oscillate with phases of the cell cycle, and bulk sequencing would miss these dynamic expression changes. Finally, the collection of GSC isolates studied here may not capture the full spectrum of GBM and GSC heterogeneity, which could impact IGC profiles and their associations with specific GBM subtypes. Therefore, building off of our current paradigm, we propose that future studies should aim to increase GSC and normal neural sample complexity. Although challenging, this could be accomplished in time through consolidation of existing RNA-seq databases or acquisition and analysis of additional samples.

IGCs may regulate GSC malignancy through several potential mechanisms. Several studies have shown that ion channels can regulate cell cycle dynamics [10], migration [11], apoptosis [12], and vascularization [44] contributing to cancer progression. The expression levels of some ion channels are regulated in tune with the cell cycle [45], and voltage-gated K^+^ channels, in particular, are known to exhibit cell-cycle-dependent fluctuations in expression or activity in non-cancerous [46] and cancerous [45] cells, which contribute to cell cycle checkpoint regulation. This supports our finding that increasing concentrations of extracellular K^+^ increase GSC and NSC viability, presumably through enhanced proliferation. Furthermore, ion channel blockers can inhibit cell cycle progression, arrest aberrantly cycling cancer cells, and inhibit tumor formation [9,45]. Ion channels can also regulate the resting membrane potential (V_m_) of cells, a process that is deregulated in cancer cells [47,48]. V_m_ is regulated with the cell cycle in non-cancerous and cancerous cells, and experimental reversal of V_m_ at these stages can arrest or stimulate the cell cycle [49,50]. Thus, the aberrant ion channel dysregulation in GBM and GSCs observed in this study could modulate V_m_ contributing to tumor progression and malignancy, and these electrophysiological changes should be examined in future studies.

There is a substantial lack of understanding of how GBM functions within the context of the normal brain environment. We propose that the neural environment and electrophysiological mechanisms play a vital role in GBM oncogenesis and maintenance. Ion channels play a crucial role in these mechanisms in multiple cancers, and increasing evidence suggests that they contribute to malignancy in GBM as well, specifically within the most malignant tumor subpopulation, the GSCs. Future studies will need to parse out the role that these ion channels play in electrophysiological interactions with the surrounding neural environment. We have identified ion channel candidates that are enriched in GSCs and are linked to GBM prognosis. Many questions still need to be addressed to understand the contribution of ion channels to GSC biology. Although connexins and transporters did not play a significant role in these findings, other molecular players, such as metabotropic receptors, should be examined in this context. Additionally, it will be critical to understand how these channels contribute to malignancy and the downstream pathways involved, particularly V_m_-related and non-current passing mechanisms. Furthermore, ion channels are known to interact with migratory, angiogenic, and apoptotic factors, which should be explored further in this setting. While choosing candidate ion channels to target based solely on expression data is informative, it will be vital to carry out functional screening assays in the future to find meaningful functional outcomes. Nevertheless, the broad enrichment of ion channel types supports the notion that electrical activity within the tumor microenvironment may regulate GBM malignancy, and much more research is needed to understand the potential reciprocal interactions between GSCs and the neural surround. Despite the study limitations described, our findings offer a starting point for exploring hypotheses of novel ion channel-based drug targeting of GSCs in vitro and in preclinical models.

## Materials and methods

### GSC culture

Human GSCs were previously isolated from resected stage IV glioma tumors (S1 Table) [33,51–56]. Non-tumor neural cell lines included: human fetal cortical neural stem cells (NSC-CB660), v-myc immortalized brainstem NSCs (NSC-VM; ReNcell, EMD Millipore), c-myc immortalized cortical NSCs (NSC-CX; ReNcell, EMD Millipore), normal human astrocytes (NHA; StemCell Technologies), and Ras-V12 infected NHAs (NHA-RAS) [57] (S1 Table). For RT-qPCR, MTT viability, and siRNA knockdown assays, cells were newly thawed from frozen stocks and maintained in culture over multiple passages using previously published protocols [3,55]. Cells were grown as adherent cultures on flasks coated with Natural Mouse Laminin (10 ng/ml; Thermo Fisher Scientific, #23017–015) in Human NeuroCult NS-A Proliferation Kit (StemCell Technologies, #05751), Heparin sodium salt (2 mg/ml, Sigma-Aldrich, #H3149), Antibiotic-Antimycotic (Thermo Fisher Scientific, #15240–062; 10,000 units/ml of penicillin, 10,000 μg/ml of streptomycin, and 25 μg/ml of Fungizone Antimycotic), supplemented with human recombinant EGF (10 ng/μl; Peprotech, #AF-100-15) and bFGF (10 ng/μl, Stemgent, #03–0002). GSCs were maintained at low passage number and passaged at 80–90% confluence (approximately every 3–4 days) with StemPro Accutase Cell Dissociation Reagent (Thermo Fisher Scientific, #A11105-01); cells were seeded at 4–5×10^5^ cells per 25 cm^2^ flask. To assess overall similarity between samples, cell isolates were authenticated by exome or RNA sequencing analysis, which included non-supervised clustering, principle component analysis, and differential expression analysis using DESeq2 [58].

### RNA sequencing and molecular subtyping

RNA sequencing was carried out as previously described [27]. Briefly, sequencing was performed using an Illumina HiSeq 2000 in Rapid Run mode and employed a paired-end, 50 base read length (PE50) sequencing strategy. RNA-seq reads were aligned to the UCSC hg19 assembly using Tophat2 [59] and counted for gene associations against the UCSC genes database with HTSeq [60]. All data was combined and normalized using a trimmed mean of M-values (TMM) method from the R package, edgeR [61]. Sequencing data can be accessed at Sequence Read Archive SRP092795 and NCBI Gene Expression Omnibus under GSE89623. Molecular classifications were determined according to a previous report [27]. For downstream analyses, replicates (2–3 per cell isolate) were averaged to assign a single CPM value to each sample. GSEA analysis [62,63] was performed using a Signal2Noise metric for ranking genes. GSC isolates were classified by molecular subtype according to gene expression signatures produced by The Cancer Genome Atlas [26,64]. Our isolates were clustered using 770 of these genes using a Manhattan distance complete-linkage method, and centroids were computed as the median expression of each gene across the core TCGA samples [26]. Each GSC sample replicate was compared against the centroids using Single Sample Predictor (SSP) method [65]. In addition, samples were assigned to GBM subtypes by maximizing the Spearman rank based correlation between expression of new samples and GBM subtype centroids. Each replicate was assigned separately and then the consensus was used to assign a final classification. For hierarchical clustering, the clustergram function in the bioinformatics toolbox of MATLAB (v. R2015b, MathWorks) was run using the euclidean distance metric and unweighted average distance linkage method.

### Exome sequencing

Exome sequencing and preprocessing were performed at the Genome Core Facility of Mount Sinai School of Medicine. Whole genome amplified was used for exome sequencing. Whole-exome capture libraries were constructed using ligation of Illumina adaptors. Each captured library was then loaded onto the HiSeq 2500 sequencing platform. Exome sequence preprocessing and analysis were performed using standard pipelines recommended by the Genome Analysis Toolkit (GATK) [66]. Three GSC cell lines were aligned independently. For each sample, the reads were aligned to NCBI build 37 (hg19) human reference sequence using BWA (http://bio-bwa.sourceforge.net) [67], and duplicated were marked using Picard (http://broadinstitute.github.io/picard/). Local realignment around indels and base recalibration process were performed ending in an analysis-ready BAM file for each cell line. Mutation detection and annotation were performed at the Genome Core Facility of Mount Sinai School of Medicine as follows. For each sample, GATK was used to detect all variants that differed from a reference genome. Variants identified were annotated using the snpEff software [68]. The variants were filtered in four steps according to a previous study [69]. First, the variants with low allelic fraction were excluded. The allelic fraction was calculated for each detected variant per cell line as a fraction of reads that supported an alternative allele (e.g. different from the reference) among reads overlapping the position. Only reads with allelic fractions above 0.25 were used in the downstream analysis. Additionally, the variants that were detected as common germline variants were excluded. Variants for which the global allele frequency (GAF) in dbSNP138 or allele frequency in the NHLBI Exome Sequencing Project (http://evs.gs.washington.edu/EVS, data release ESP2500) was higher than 0.1% were excluded from further analysis. Furthermore, variants detected in a panel of 278 whole exomes sequenced at the Broad Institute as part of the 1000 Genomes Project were excluded from further analysis. Finally, the variants with low quality (e.g. insufficient read depth and insufficient genotype quality) were filtered with the variant quality score tools. We selected high-confident mutations by their annotation obtained from snpEff. We filtered silent mutations and extracted high and moderate impact of mutations, including non-synonymous, nonsense, frame shift, and codon insertion/deletion mutations. Exome sequencing data can be accessed at NCBI Sequence Read Archive SRP09879 under BioProject PRJNA369688.

### TCGA and Ivy GAP GBM databases

TCGA level 3 GBM data for U133A microarrays (539 samples), along with the corresponding clinical data, was downloaded from the TCGA Data Portal (https://tcga-data.nci.nih.gov/tcga/). The data was imported into an R environment. Samples for which there was no clinical data relating to time of death were excluded resulting in 525 total samples. Expression levels for the gene of interest were then pulled, and samples were sorted low to high by expression level; the top and bottom expressing samples were identified using the quantile function (0.10 and 0.90, respectively). Kaplan-Meier curves were generated using the survfit function in the survival library of R comparing the low and high cohorts. For Ivy GAP analyses, FPKM values and sample information were downloaded from the Ivy GAP website (glioblastoma.alleninstitute.org). FPKM values were averaged across replicates within regions to generate single values for each sample within a particular region.

### RNA isolation and real-time qPCR

RNA was extracted from cell cultures every two weeks during the exponential growth phase; RNA was isolated from 0.5–1.0×10^6^ cells. Total RNA was prepared using RNeasy Mini Kit with DNase I (Qiagen). RNA concentration and quality (A260/A280) was measured using a NanoDrop 1000 Spectrophotometer (Thermo Scientific). cDNA was synthesized from 2 μg of RNA using iScript Reverse Transcription Supermix for RT-qPCR (Bio-Rad). Quantitative real-time PCR was performed with iTaq Universal SYBR Green Supermix (Bio-Rad) on an Applied Biosystems 7300 Real Time PCR System. Reactions were performed in triplicate and values normalized to *ACTB* (β-Actin). RT-qPCR primer sequences were designed to span exon-exon boundaries and are listed in S3 Table.

### Drugs

Drugs used for this study included: tetrodotoxin (TTX, voltage-gated Na^+^ channel blocker, Tocris, #1078), tetraethylammonium hydroxide (TEA, non-inactivating K^+^ channel blocker, Sigma-Aldrich, #T6393), 4-Aminopyridine (4-AP, transient/A-type K^+^ channel blocker, Sigma-Aldrich, #275875), 3-((±)2-carboxypiperazin-4yl)propyl-1-phosphate (CPP, NMDA receptor antagonist, Tocris, #0173), 6-Cyano-7-nitroquinoxaline-2,3-dione (CNQX, AMPA/kainate receptor antagonist, Alomone Labs, #C-140, diluted in DMSO), ω-Conotoxin MVIIC (N-, P/Q-type Ca^2+^ channel blocker, Alomone Labs, #C-150), cadmium chloride (CdCl_2_, non-selective voltage-gated Ca^2+^ channel blocker, Sigma-Aldrich, #C3141), Gabazine (GABA_A_ receptor blocker, Tocris, #1262), Picrotoxin (PTX, GABA_A_ receptor blocker, Sigma-Aldrich, #P1675, diluted in DMSO). KCl was added at indicated concentrations to growth media, which had undisclosed levels of K^+^.

### MTT viability assay

Cells were harvested and seeded on laminin-coated 96-well plates at a density of 10^4^ cells per well. The following day, cells were attached and compounds were added to the media at indicated concentrations. Untreated conditions received DMSO or water, as appropriate. Seventy-two hours after compounds were added, the Vybrant MTT Cell Proliferation Assay Kit (Thermo Fisher Scientific, #V13154) was performed according to manufacturer’s instructions. 10 μl per well of 12 mM stock MTT compound (dissolved in sterile PBS) was added to wells and incubated for 4 hours at 37°C. Viable cells reduced MTT into purple formazan crystals, which was solubilized in a solution of SDS-0.1 M HCl added to wells for 4 hours at 37°C. Absorbance at 570 nm was read at 37°C using a SpectraMax 190 Gemini Microplate Reader. Each condition was run in triplicate. Fluorescence arbitrary units (AU) were subtracted from background levels, averaged across triplicate wells, and normalized to untreated wells.

### siRNA-mediated knockdown

Human DsiRNA kits (Integrated DNA Technologies) consisting of three predesigned DsiRNAs against selected IGCs (*GRIA3*, *KCNB1*, and *SCN8A*) were selected along with a Scrambled Negative Control DsiRNA. Cells were plated on laminin-coated tissue culture plates for CellTiter-Glo Luminescent Cell Viability (Promega; 96-well) or RT-qPCR (6-well) assays 24 hours before transfection. Cells were 30–40% confluent at the time of transfection. For RT-qPCR experiments, 100 pmol RNA was transfected per well, and 2, 5, or 20 pmol RNA was transfected per well for the CellTiter-Glo assay. Antibiotics and antimycotics were removed from media, and transfection was carried out with Lipofectamine RNAiMAX Transfection Reagent (Thermo Fisher Scientific) and Opti-MEM Reduced Serum Medium (Thermo Fisher Scientific) according to the manufacturer’s instructions. Negative controls for all experiments included non-transfection wells (not shown) and wells transfected with scrambled DsiRNA. RNA isolation and RT-qPCR was carried out 24 hours after transfection, and the CellTiter-Glo assay was performed 72 hours after transfection according to methods outlined by the manufacturer.

### Statistics

Statistics were performed using GraphPad Prism 6, R version 3.2.3, or MATLAB (R2015b, MathWorks). Comparisons between two groups were tested for significance with the Mann-Whitney test. For multiple comparisons, the Kruskal-Wallis test was used to compare across molecular subtypes. Kaplan-Meier plots were created using the R package “survival” with the survfit function, and the log-rank (Mantel-Cox) test was used to test for significance of the Ivy GAP data. Two-way ANOVA with repeated measures was used for data with multiple drug concentrations (MTT and CellTiter-Glo assays); runs with missing values were excluded from statistical testing but included on plots.

## Supporting information

S1 Table Table of cell isolates used in this study. Only gene mutations in RTK/RAS/PI(3)K, p53, and RB pathways in which the mutation was present in at least three samples were included.
(EPS)

S2 Table RNA sequencing CPM values for all genes and ion channel gene set. Ion channel gene list derived from guidetopharmacology.org [22].
(XLSX)

S3 Table Real-time qPCR primer sequences. (EPS)

S4 Table GSC-enriched ion channel details. Top 25 IGCs identified by differential enrichment analysis grouped by functional relatedness.
(EPS)

S1 Fig Schematic summarizing study’s design. (EPS)

S2 Fig Gap junction protein expression values. Heat map (unranked) of log_2_ fold change values of all connexins and pannexins in GSCs compared to control NSCs/NHAs. Each column represents log_2_ fold change values (compared to averaged values across NSCs/NHAs) from a distinct cell isolate after averaging triplicate CPM values.
(EPS)

S3 Fig Expression pattern of IGCs in non-tumor neural cell types. Expression levels of top 25 IGC candidates were examined in several human non-tumor neural cell types from RNA sequencing data described by Zhang et al. [25] and downloaded from http://www.brainrnaseq.org. IGCs were specifically enriched in neurons, astrocytes, microglia, or across multiple classes. Three IGCs are listed that had low abundance (mean expression in at least one cell type was not ≥1 FPKM). Bars, mean FPKM values ± SEM. GBM-A, GBM/peri-tumor astrocytes; SH-A, sclerotic hippocampi astrocytes; F-A, fetal astrocytes; M-A, mature astrocytes; N, neurons; O, oligodendrocytes; M, microglia; E, endothelial cells; WC, whole cortex.
(EPS)

S4 Fig Expression heat map of ion channel families by tumor region. FPKM values of ion channels partitioned by ion channel family and GBM tumor region (Ivy GAP RNA-seq database).
(EPS)

S5 Fig Summary of results relating to IGCs reported in this study. Top 25 IGCs and associated analyses. R/R/P, RTK/RAS/PI(3)K pathway; TP53, TP53 pathway; RB, RB pathway.
(EPS)
